# Computational Discovery of Novel SGLT2 Inhibitors from Eight Selected Medicine Food Homology Herbs Using a Multi-Stage Virtual Screening Pipeline

**DOI:** 10.3390/ph19020246

**Published:** 2026-01-31

**Authors:** Zeyu Chen, Kaiqi Tan, Yi Shi, Muchong Liu, Lang Yi, Tongxi Chen, Yunlong Bai

**Affiliations:** 1 Guangdong Provincial Laboratory of Traditional Chinese Medicine, Key Laboratory of Chinese Medicinal Resource from Lingnan, Ministry of Education, Guangzhou University of Chinese Medicine, Guangzhou 510006, China; czy17818521026@163.com (Z.C.); 20241110119@stu.gzucm.edu.cn (K.T.); liumichong0314@163.com (M.L.); langyi@gzucm.edu.cn (L.Y.); chentongxi02@163.com (T.C.); 2 Key Laboratory of Chronic Disease Prevention and Treatment in Traditional Chinese Medicine, Guangdong Provincial Higher Education Institutions, Guangzhou 510006, China; 3 Independent Researcher, Hangzhou 310024, China; 2011shiyi@sina.com

**Keywords:** SGLT2 inhibitors, machine learning, medicine food homology herbs, Isoononin, Ononin, molecular dynamics simulation

## Abstract

**Background/Objectives**: Sodium-glucose co-transporter 2 (SGLT2) inhibitors are essential antidiabetic medications. However, their side effects warrant careful consideration. The search for novel SGLT2 inhibitors with high affinity remains an ongoing endeavor. Medicine food homology (MFH) herbs show promise for drug development due to their nutritional and medicinal value. **Methods**: This study aims to address the shortcomings of existing virtual screening models for SGLT2 inhibitors by optimizing feature selection and integrating multidimensional molecular fingerprints. Subsequently, an integrated virtual screening pipeline is constructed to identify potential SGLT2 inhibitors from eight selected MFH herbs. **Results**: The results indicate that the optimal model (LightGBM and RF) achieved an accuracy of 0.97 and an AUC of 0.98. Following rigorous filtering, a total of 44 potential SGLT2 inhibitors were identified, among which, Isoononin (from Gancao) and Ononin (from Huangqi, Gegen, and Gancao) exhibit favorable drug likeness and safety. Molecular docking demonstrate that both compounds can effectively bind to the SGLT2 active site, establishing stable hydrophobic interactions with critical residues such as Phe98 and Phe453. Furthermore, molecular dynamics simulations confirm the stability of the interactions between the two compounds and SGLT2. **Conclusions**: This study significantly enhances the accuracy and stability of SGLT2 inhibitor virtual screening models by addressing deficiencies in structural characterization and feature selection. It provides candidate molecules for the development of novel SGLT2 inhibitors and offers new scientific evidence for the application of MFH herbs in the prevention and treatment of chronic metabolic diseases.

## 1. Introduction

Diabetes is a chronic metabolic disease characterized by hyperglycemia, and its prevalence continues to rise globally, becoming a serious public health issue. According to the International Diabetes Federation’s Diabetes Atlas 11th edition 2025 report, the number of adults aged 20–79 with diabetes worldwide reached 589 million in 2024, accounting for 11.1% of the total population. This figure is projected to increase to 853 million by 2050, representing a 45% rise [1]. Diabetic patients are susceptible to serious complications, including cardiovascular diseases, kidney diseases, and neuropathy [2]. Among these, cardiovascular complications are one of the leading causes of mortality in diabetic patients. Statistics indicate that approximately 3.4 million people died from diabetes globally in 2024, which accounts for 9.3% of global deaths, with 50–70% of these fatalities related to cardiovascular complications [1]. Therefore, the threat posed by diabetes and its complications to human health is increasingly severe, underscoring the urgent need for safe and effective treatment methods.

SGLT2 inhibitors represent a significant breakthrough in diabetes treatment in recent years. Their primary function is to inhibit the reabsorption of glucose in the renal tubules, thus promoting urinary glucose excretion and lowering blood sugar levels. Under normal physiological conditions, the SGLT2 protein is responsible for the reabsorption of approximately 90% of glucose from the renal filtrate. However, in patients with type 2 diabetes mellitus (T2DM), the overactivation of SGLT2 leads to increased glucose reabsorption, exacerbating hyperglycemia [3,4]. By specifically blocking this process, SGLT2 inhibitors effectively reduce blood glucose levels. Clinical studies have confirmed that these drugs also offer additional cardiovascular and renal protective benefits, significantly reducing the risk of cardiovascular events and the progression of kidney disease [5,6,7].

**Figure 1:**
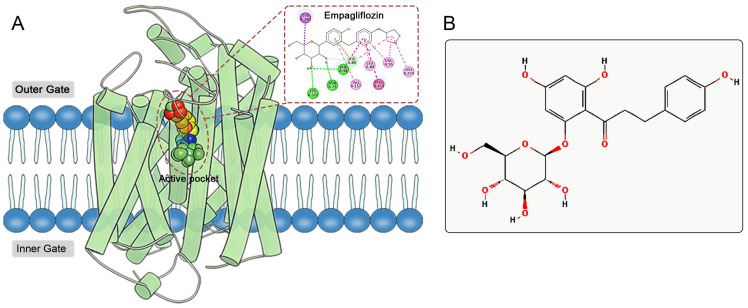
(**A**) Active pocket and ligand binding site of SGLT2. Empagliflozin serving as a model ligand to illustrate its interaction pattern with key amino acid residues, which are highlighted. (**B**) 2D structure of phlorizin.

The active site of SGLT2 is located within the hydrophobic pocket of the protein’s transmembrane region (Figure 1A). This hydrophobic pocket comprises several key amino acid residues, primarily including His80, Phe98, Val95, Leu84, Phe453, and Leu274 [8,9]. Mechanistically, SGLT2 inhibitors occupy the active site, forming hydrogen bonds and hydrophobic interactions with these key residues, which reduces glucose binding to SGLT2, thereby inhibiting its glucose transport function [8,9]. Furthermore, the inhibitory effects of SGLT2 inhibitors on sodium reabsorption indirectly enhance myocardial energy metabolism and cardiac function [10]. Recent research has indicated that SGLT2 inhibitors may provide cardioprotective benefits by activating the AMPK pathway, suppressing the NLRP3 inflammasome, and lowering ROS production, independent of their glucose-lowering properties [11,12,13,14]. Their role in promoting the production and use of ketone bodies optimizes the myocardial substrate supply, boosts mitochondrial efficiency, and mitigates ventricular remodeling [15,16,17]. These multi-target mechanisms demonstrate significant advantages in treating heart failure among diabetic populations.

Phlorizin, a naturally occurring compound derived from the bark of apple tree roots, serves as a prototype for SGLT2 inhibitors (Figure 1B) [18]. By utilizing the pharmacophore of phlorizin, scientists have developed several SGLT2 inhibitors with improved pharmacological characteristics, including Dapagliflozin, Empagliflozin, and Canagliflozin [19]. Compared to phlorizin, these synthetic inhibitors demonstrate greater selectivity for SGLT2 and extended half-lives in the body, making them preferred options for treating T2DM and its associated complications. However, existing SGLT2 inhibitors still encounter challenges related to potential adverse reactions. Data from the FDA Adverse Event Reporting System (FAERS) from 2012 to 2022 indicate that the adverse reactions of SGLT2 inhibitors exhibit distinct drug-specific characteristics [20]. Empagliflozin is significantly associated with reports of elevated blood glucose, nausea, and dizziness, while adverse reaction reports for Dapagliflozin predominantly involve weight loss. In contrast, Canagliflozin is linked to a variety of high-risk adverse reactions, including diabetic ketoacidosis, toe amputation, acute kidney injury, osteomyelitis, fungal infections, and urinary tract infections, with reports of osteomyelitis and toe amputation being particularly notable. These adverse reactions not only limit medication options for certain patients but also pose challenges for clinical safety in medication use. In this context, there is an urgent need to develop new SGLT2 inhibitors that effectively combine efficacy and safety.

MFH herbs, as an important part of traditional medicine and modern healthcare, not only possess nutritional value but also exhibit medicinal properties, providing a rich resource of natural compounds for the exploration of new SGLT2 inhibitors that are both safe and bioactive. Importantly, MFH herbs, such as Huangqi, Maidong, Gegen, and Gancao, are rich in active components like flavonoids, saponins, terpenes, and polysaccharides. The polysaccharide content in Maidong can exceed 22.80% [21], while in Huangqi, it can reach up to 15% [22]. Flavonoid compounds in Gegen can constitute more than 8% of the total mass, encompassing over 40 types, including puerarin, soybean flavonoids, and daidzein [23]. The flavonoid content in Gancao is approximately 10.1%, comprising glycyrrhizin, isoliquiritigenin, liquiritin, and isoliquiritin [24]. Concurrently, these compounds have established a mature extraction technology system, where extraction methods must be selected based on the polarity and chemical stability of the target compounds. Traditional solvent extraction and hot reflux methods are suitable for components with high stability and clear polarity. For instance, the more polar glycosides in flavonoids can be extracted using hot reflux with water–ethanol [25], whereas the less polar flavonoid aglycones can be extracted using organic solvents such as chloroform [26]. Given their strong water solubility and good thermal stability, hot water extraction has become a conventional choice for polysaccharides [27]. Modern technologies, such as ultrasound, microwave, enzyme-assisted extraction, and supercritical fluids, focus on enhancing efficiency. Heat-sensitive flavonoid components can be extracted using ultrasound, microwave, or Semi-Bionic Extraction (SBE) methods to prevent structural damage [28,29]. High-speed counter-current chromatography can achieve the direct purification of crude extracts [30]. Polysaccharides are enhanced in solubility through ultrasound-enzyme synergy and high-pressure pulsed electric field technology, while maintaining the integrity of macromolecular structures [31,32]. The rich reserves of active substances and advanced extraction technologies provide a high-quality library of natural compounds and a material basis for the subsequent exploration of novel SGLT2 inhibitors.

Computer-aided virtual screening has become an important method for discovering novel SGLT2 inhibitors. The similarity of ligand structures [33] and the affinity of receptor structures [33,34] are the primary methods utilized in the virtual screening of SGLT2 inhibitors. In recent years, the application of ML has significantly enhanced the efficiency of drug virtual screening. Moinul et al. (2022) employed Bayesian classification (ECFP_6 fingerprints) and recursive partitioning (FCFP_6 fingerprints) to perform key feature selection on 224 SGLT2 inhibitors [34], and constructed QSAR models using nine ML algorithms, ultimately screening 14 potential SGLT2 inhibitors from the FDA drug database [34]. Feature selection is a crucial step in machine learning that directly impacts the model’s generalization ability and the accuracy of predictions. Existing models utilize correlation analysis for feature selection. While this method effectively identifies molecular features related to activity, its capacity to filter sparse and redundant features in high-dimensional data is limited. Such features can easily contribute to model overfitting. Therefore, removing low-variance features through ANOVA and eliminating redundant features via Pearson correlation analysis can effectively reduce noise interference in the feature space, improving the quality and interpretability of the feature subset, thereby enhancing the robustness and predictive accuracy of the model.

**Figure 2:**
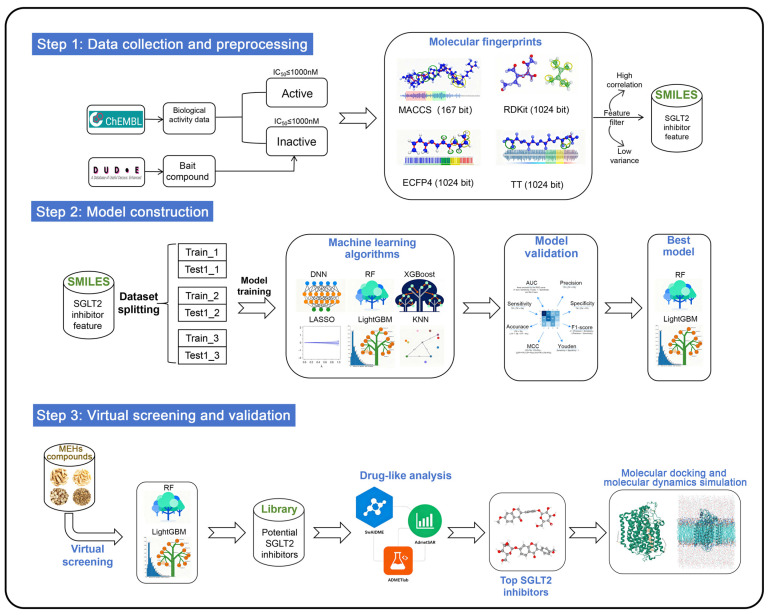
Flow chart of identification of novel SGLT2 inhibitors.

In addition, different molecular fingerprints represent compound structures in distinct ways, which may lead to discrepancies in the assessment of SGLT2 activity when models are constructed using different fingerprints. However, current virtual screening studies on SGLT2 inhibitors rely exclusively on a single type of molecular fingerprint to describe compound structural features. The integration of multiple molecular fingerprints into ML models can effectively consolidate structural information of compounds at various levels, thereby compensating for the limitations of a single fingerprint in characterization capabilities. MACCS fingerprints effectively represent the geometric and topological features of molecular structures [35], while RDKit fingerprints encode the linear arrangements of atoms and bonds [36]. Extended connectivity fingerprints radius 2 (ECFP4) utilize iterative hashing to represent cyclic atomic environments [37]. Topological torsion fingerprints (TT) provide insights into torsional and topological aspects of molecular graphs by capturing sequences of connected atomic environments [38]. This study constructs an integrated virtual screening pipeline by incorporating MACCS, RDKit, ECFP4, and TT to identify potential SGLT2 inhibitors from a compound library of eight MFH herbs. The complete research methodology is illustrated in Figure 2.

This study’s innovation lies in the development of an integrated virtual screening pipeline for SGLT2 inhibitors, along with the identification of natural compounds with potential SGLT2 inhibitory activity derived from MFH herbs. Compared to existing models, this study enhances the model’s generalization ability and stability by optimizing feature selection and integrating multidimensional molecular fingerprint. Furthermore, this research presents new candidate molecules for the development of novel SGLT2 inhibitors and provides new scientific evidence supporting the application of MFH herbs in the prevention and treatment of chronic metabolic diseases.

## 2. Results

### 2.1. Compound Similarity Analysis

**Figure 3:**
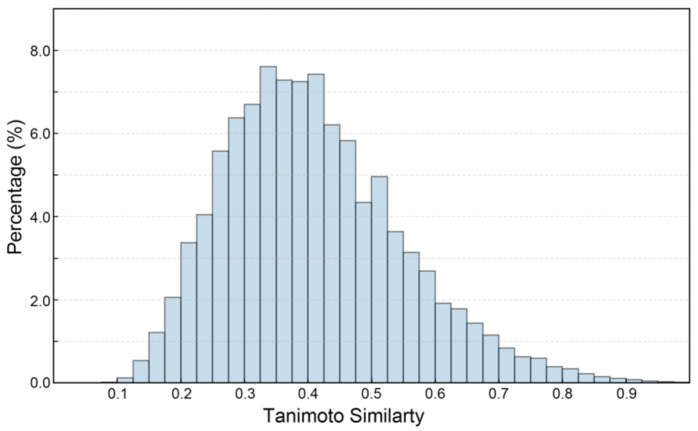
The Tanimoto similarity histogram of SGLT2 inhibitor pairs.

Figure 3 presents the Tanimoto similarity histogram of compound pairs in the constructed SGLT2 dataset (ChEMBL and DUD-E database), reflecting the structural diversity of the dataset. The variety in dataset structures is essential for creating ML models that are both robust and capable of generalization. This is especially true in drug discovery, where chemical diversity improves the model’s ability to identify new scaffolds [39]. As shown, the SGLT2 inhibitors shows a broad range of Tanimoto coefficients, reflecting significant structural variability among the compounds in the dataset (Figure 3). Notably, 75.92% of the compound pairs demonstrate low to moderate similarity (Tanimoto coefficient < 0.5), indicating that the dataset covers a wide chemical landscape instead of being confined to a limited number of scaffold types [40]. This structural diversity is important as it reduces the chances of overfitting during model training and enhances the potential for identifying novel SGLT2 inhibitors. Additionally, this chemical variety strengthens the robustness of classification tasks, allowing the predictive model to generalize effectively to new chemical entities.

### 2.2. Dataset Segmentation and Chemical Spatial Distribution

**Figure 4:**
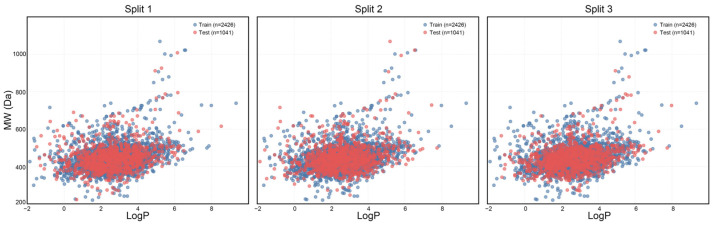
Chemical spatial distribution of the training and test sets in the three dataset splits.

Examining the distribution of chemical space within both the training and testing datasets is essential for evaluating the effectiveness of the dataset partitioning approach. In this research, we utilized the octanol-water partition coefficient (LogP) and molecular weight (MW) to create a two-dimensional visualization of the chemical landscape, which allows for the assessment of the physicochemical variety between the training and testing datasets (Figure 4). The findings reveal that across three distinct partitioning techniques, blue markers denote compounds in the training dataset, whereas red markers indicate those in the test dataset. The overlap in the LogP-MW two-dimensional representation suggests that both datasets provide comprehensive coverage of the chemical space. Importantly, compounds with extreme characteristics (LogP > 7 or MW > 800) are found in both datasets, demonstrating that the partitioning method successfully preserves critical boundary information within the chemical space, thereby preventing shifts in data distribution due to segmentation. This balanced distribution supports the model’s capacity to learn a wide range of molecular characteristics during training, minimizing the risk of overfitting to the edges of the chemical space and simultaneously improving its predictive accuracy for unfamiliar compounds.

### 2.3. Model Performance Analysis

**Figure 5:**
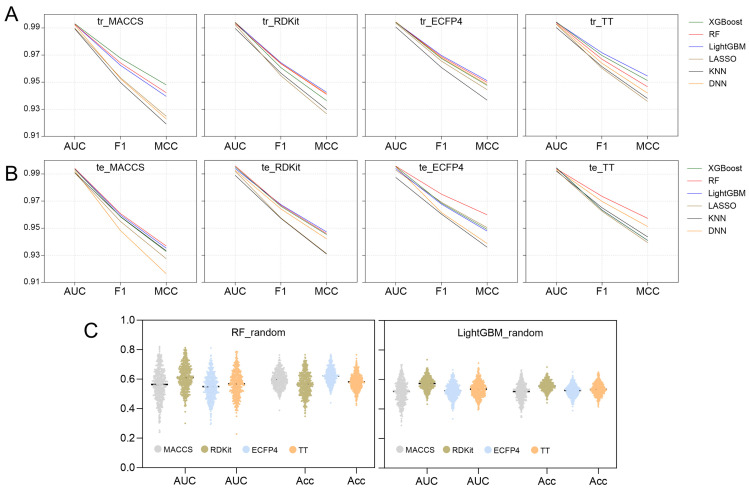
Comparison of AUC, F1 scores and MCC between different model (**A**) training sets and (**B**) testing sets. (**C**) AUC and accuracy of the Y-randomization model.

AUC, F1 score, and MCC play a crucial role in assessing the overall performance of models [41]. Figure 5 provides a summary of these parameters across all 24 models, leading to several key insights: (1) Each model demonstrates strong fitting and generalization abilities for the binary classification task, with AUC, F1 scores, and MCC values all surpassing 0.98, 0.94, and 0.91, respectively. This suggests that they are all capable of effectively identifying active compounds across various molecular fingerprint formats. (2) When comparing molecular fingerprint descriptors, TT and ECFP4 show a slight edge over RDKit and MACCS, indicating that topological fingerprints and ring sequence characteristics are more adept at capturing the local structural details of molecules. (3) Among the six algorithms assessed, the LightGBM model exhibits a marginally better fitting performance, especially with TT, ECFP4, and RDKit. However, the RF model displays a somewhat enhanced generalization capability, with its performance metrics on the test set ranking highly for TT, ECFP4, and MACCS.

In conclusion, both the LightGBM models and RF models demonstrate good performance across various molecular characteristics. The LightGBM model excels in fitting high-dimensional fingerprint data (RDKit, ECFP4, and TT) due to its effective feature selection and ensemble learning capabilities. Meanwhile, the RF model exhibits excellent generalization ability, particularly with the MACCS, ECFP4, and TT types. Table 1 summarizes the performance metrics for both models. The precision, sensitivity, specificity, accuracy, and Youden index of each model for the four types of fingerprints all exceed 0.92, indicating that the classification ability of each model in identifying active compounds is highly reliable.

To assess the genuine predictive power of the model, an internal validation was performed utilizing Y-randomization tests on the top-performing LightGBM and RF models to determine the presence of any random correlations. The results of 500 iterations, including AUC values and accuracy distributions, are illustrated in Figure 5C. A combined analysis of Table 1 and Figure 5C demonstrates that the optimal model’s AUC values and accuracy significantly surpass those of the random model. This suggests that the predictions made by the LightGBM and RF models are a result of effective learning of molecular structural characteristics rather than chance. Consequently, LightGBM and RF models can be applied for virtual screening.

### 2.4. Compound Activity Prediction

**Figure 6:**
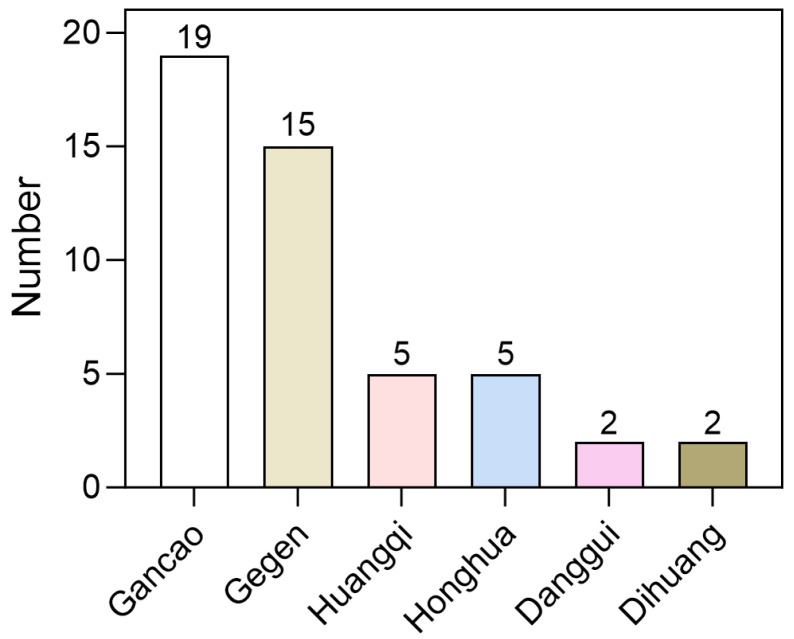
Distribution of 44 high-confidence candidate compounds in each herbs.

We utilized LightGBM and RF to create a set of four distinct fingerprint models for assessing 858 compounds derived from MFH herbs. The outcomes of this virtual screening are presented in Supplementary File S3. The analysis revealed that 204 compounds were predicted as potential SGLT2 inhibitors. However, the majority of compounds were approved by only one or two models. To enhance the reliability of the results, only those compounds recognized as active by all four models were selected, resulting in a final list of 44 potential inhibitors (Supplementary File S4). As shown in Figure 6, these compounds are primarily distributed among herbs such as Gegen, Gancao and Huangqi, with Gegen and Gancao exhibiting a higher distribution ratio. These findings suggest that Gegen and Gancao may serve as important herbal resources rich in SGLT2 inhibitors.

### 2.5. Drug-like Properties and ADME/T Analysis

#### 2.5.1. Drug-like Analysis

To identify candidate compounds for SGLT2 inhibitors with promising drug development potential, we evaluated drug-like properties based on the classical Lipinski’s Rule of Five (MW ≤ 500, HBD ≤ 5, HBA ≤ 10, LogP ≤ 5) and the Verber Rule (Rotatable Bonds ≤ 10, TPSA ≤ 140). A total of 44 compounds, identified with high SGLT2 inhibitory potential through LightGBM and RF models, were assessed for their drug-like characteristics. The results indicate that four compounds fully satisfy the aforementioned six drug-like criteria, thereby providing a solid foundation for drug development (Table 2). The drug-like analysis results for the remaining compounds are detailed in Supplementary File S4. All indicators of the four high-potential compounds demonstrated excellent balance, with only slight variations in the LogP value, indicating a significant similarity in their molecular structures.

#### 2.5.2. ADME Analysis

Based on the predictions from SwissADME (V2017), the four compounds exhibit similar ADME characteristic profiles (Table 3). All compounds demonstrate high gastrointestinal absorption, indicating good oral bioavailability; however, none are able to penetrate the blood–brain barrier, which helps avoid potential central nervous system side effects. Nonetheless, all compounds are predicted to be CYP3A4 inhibitors, suggesting a potential risk of drug–drug interactions when used in combination. CYP3A4 is one of the major enzyme systems within the CYP450 family, responsible for the metabolism of the vast majority of exogenous chemicals, including drugs [42]. Therefore, co-administration with other drugs metabolized by CYP3A4 may inhibit the metabolism of those drugs, resulting in abnormal increases in plasma concentrations and consequently increasing the risk of toxic reactions. In terms of distribution and transport, ononin and formononetin-7-glucoside have been identified as P-glycoprotein substrates. P-glycoprotein is an important efflux transporter widely expressed in tissues such as the intestine, blood–brain barrier, liver, and kidneys; its primary function is to pump substrate drugs out of cells, thereby limiting their absorption and distribution [43]. Thus, the absorption and tissue distribution of ononin and formononetin-7-glucoside in vivo may be influenced by P-glycoprotein, potentially limiting their bioavailability.

In summary, the four compounds exhibit high absorption and predominantly peripheral distribution characteristics in terms of ADME properties. The primary risks are concentrated on CYP3A4-mediated metabolic interactions and the P-glycoprotein efflux effects of some compounds.

#### 2.5.3. Toxicity Analysis

We conducted a preliminary toxicological risk assessment of four high-potential compounds (Table 4). The analysis results indicate that all compounds exhibited low hERG channel inhibition and low conventional human hepatotoxicity risks. The hERG channel is a crucial ion channel that regulates cardiac electrophysiological activity, and its dysfunction can lead to prolonged QT intervals in cardiomyocytes, potentially triggering life-threatening arrhythmias such as Torsades de Pointes [44,45]. Therefore, low hERG inhibition suggests that these compounds minimally interfere with cardiac electrophysiological homeostasis, indicating good cardiac safety. Additionally, the lower risk of human hepatotoxicity suggests that these compounds pose a reduced risk of hepatocyte damage at conventional doses. However, in the critical prediction of Drug-Induced Liver Injury (DILI) risk, the compounds 8-methoxy-5-o-glucoside flavone and formononetin-7-glucoside were flagged as high-risk, while Ononin and Isoononin were categorized as moderate risk. Furthermore, regarding genotoxicity and carcinogenicity, formononetin-7-glucoside received the highest risk rating (moderate AMES toxicity, high carcinogenicity), followed by 8-methoxy-5-o-glucoside flavone (moderate AMES toxicity, moderate carcinogenicity).

In a comprehensive comparison, Ononin and Isoononin exhibit relatively superior safety profiles, as both compounds received no high-risk ratings across various indicators. Therefore, we will focus on these two compounds as key subjects for further research.

### 2.6. Molecular Docking

**Figure 7:**
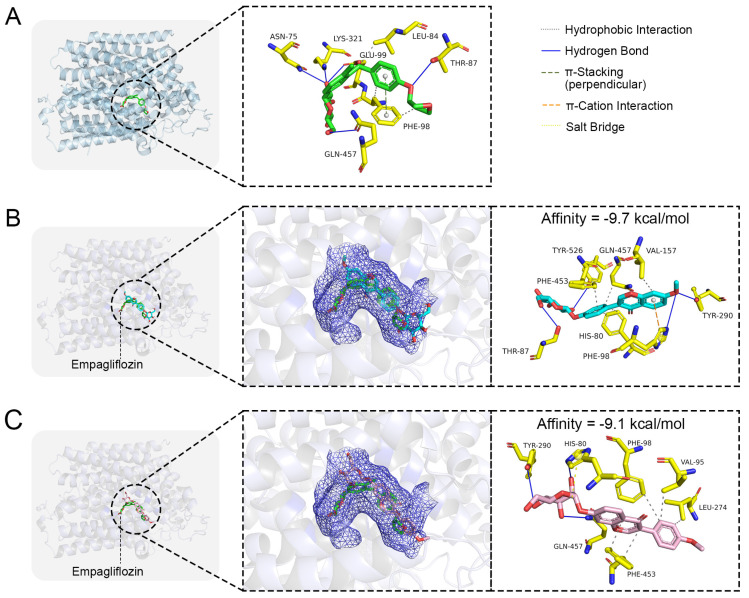
Molecular docking of small molecule ligands with SGLT2 protein. (**A**) The co-crystal structure of SGLT2-Empagliflozin. The binding conformation of (**B**) Isoononin and (**C**) Ononin in the active pocket of SGLT2.

To explore the interaction and binding characteristics of Isoononin and Ononin with the SGLT2 protein, a molecular docking study was performed utilizing the co-crystal structure of SGLT2 in complex with empagliflozin (PDB ID: 7VSI) as a reference (Figure 7A). Initially, we illustrated the binding conformation of the SGLT2 protein with the empagliflozin as recorded in the PDB database to identify the active site of the SGLT2 protein (Figure 7A). As depicted in Figure 7A, the empagliflozin’s hydroxyl group establishes hydrogen bonds with the amino acid residues Asn75, Thr87, Glu99, Lys321, and Gln457 located within the active site, while its phenyl ring participates in π-π stacking interactions with Phe98. Moreover, the hydrophobic segment of empagliflozin interacts with the side chains of Leu84 and Phe98.

The findings from molecular docking suggest that Isoononin and Ononin can both effectively attach to the active site of SGLT2 (Figure 7B,C), exhibiting binding energies of −9.7 kcal/mol and −9.1 kcal/mol, respectively. The docking conformation for Isoononin (Figure 7B) illustrates that this compound maintains its attachment through various interactions, such as hydrophobic forces, hydrogen bonds, and π-cation interactions. Specifically, the benzopyran ring participates in a π-cation interaction with His80 and establishes hydrophobic interactions with Phe98, Phe453, Gln457, and Val157. Additionally, the hydroxyl groups present on the glucoside and the benzopyran ring create a network of hydrogen bonds with Thr87, Tyr290, Tyr526, and His80, which further enhances the binding’s specificity and stability. In the case of Ononin (Figure 7C), its benzopyran ring is accommodated within the hydrophobic pocket of SGLT2, engaging in hydrophobic interactions with Val95, Phe98, Leu274, and Phe453. At the same time, its glucoside forms hydrogen bonds with His80, Gln457, and Tyr290, while also creating a salt bridge with His80, which reinforces Ononin’s binding within the active site. Despite the differences in their binding configurations, both isomers successfully occupy the active pocket, indicating strong binding affinity towards the SGLT2 protein.

### 2.7. Molecular Dynamics Simulation

**Figure 8:**
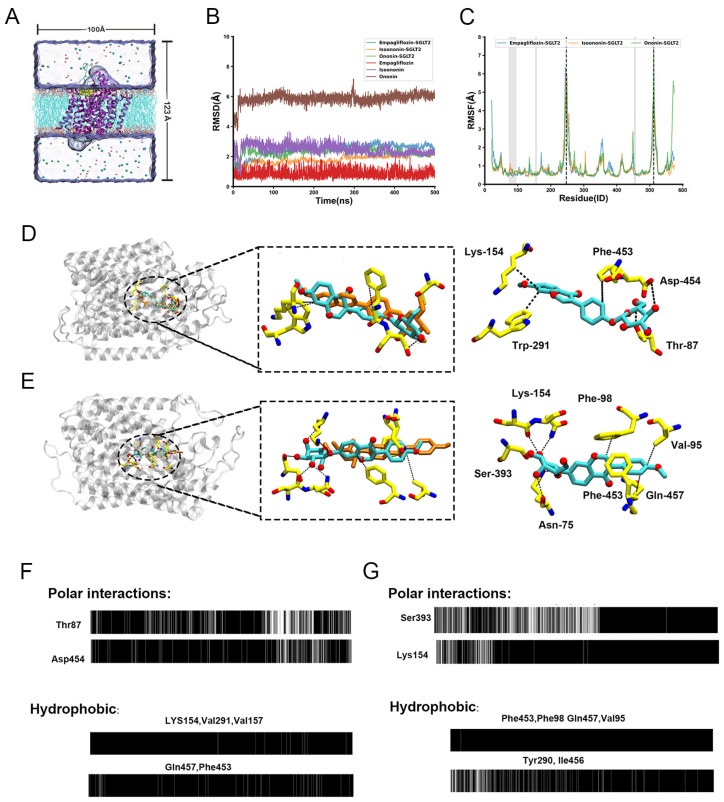
Molecular Dynamics (MD) simulations reveal the stability and interaction patterns of Isoononin and Ononin with SGLT2. (**A**) Schematic representation of the initial simulation system, showing an inhibitor-bound SGLT2 complex embedded in a hydrated POPC lipid bilayer. The initial binding pose was determined from molecular docking. (**B**) Time evolution of the Root-Mean-Square Deviation (RMSD). The RMSD of the protein Cα atoms is plotted for the SGLT2 complexes with Empagliflozin, Isoononin, and Ononin. Additionally, the RMSD of the heavy atoms for each respective inhibitor is shown, calculated after aligning to the protein backbone. (**C**) Per-residue Root-Mean-Square Fluctuation (RMSF) profiles for the Cα atoms of SGLT2 in each complex, calculated over the 500 ns simulation. The gray shaded area highlights the inhibitor binding region, while dashed lines indicate key flexible loop regions. (**D**) Schematic representation of the binding modes and predominant interactions for Isoononin (**D**) and Ononin (**E**) within the SGLT2 active site. The left panels show the binding snapshot within the protein scaffold. The central panels illustrate the superimposition of the initial inhibitor pose calculated by AutoDock (orange carbons) with the representative MD simulation snapshot (cyan carbons). Key residues forming stable interactions are highlighted in the right panels, Black dashed lines denote specific intermolecular interactions, including hydrogen bonds and hydrophobic contacts. (**F**,**G**) Barcode representation of the timelines of typical protein-inhibitor contacts for Isoononin (**F**) and Ononin (**G**). The upper panels show the persistence of specific hydrogen bonds, and the lower panels show hydrophobic interactions over the 500 ns trajectory. A black bar indicates that the interaction is present in that simulation frame.

To assess the stability of the docked poses and explore the binding dynamics, we performed 500 ns all-atom MD simulations for each SGLT2-inhibitor complex (SGLT2 bind with Isoononin, Ononin and FDA-approved drug Empagliflozin). The overall conformational stability of the protein was evaluated by calculating the root-mean-square deviation (RMSD) of the backbone relative to the initial structure (Figure 8B). In all three systems, the protein backbone RMSD equilibrated quickly and remained stable at approximately 2 Å, indicating that the overall fold of SGLT2 was well-maintained.

In contrast, the RMSD calculated for the inhibitors revealed distinct positional stabilities. As the control, Empagliflozin was exceptionally stable, with its RMSD remaining below 1.5 Å, confirming a persistent binding pose. Isoononin also demonstrated stable binding, with its RMSD equilibrating at a slightly higher value of ~2.5 Å. Notably, Ononin underwent a significant shift within the first 20 ns, with its RMSD rapidly increasing to ~6 Å before stabilizing at a more favorable binding mode within the binding pocket. This behavior suggests that despite the instability of Ononin’s initial docked pose which was relatively close to the optimal binding conformation, it could rapidly adjust to a more stable state within the pocket. This behavior suggests that despite the instability of Ononin’s initial docked pose, which deviated from the optimal binding conformation, it could rapidly adjust to a more stable state within the pocket.

To characterize the flexibilities of the protein, we calculated per-residue root-mean-square fluctuations (RMSF), as shown in Figure 8C. The RMSF profiles were highly consistent among the three systems, with most residues exhibiting low fluctuations (<2 Å). This was evident for residues constituting the binding pocket (e.g., 74–95, 154–157, 453–457; highlighted in gray), which confirms that the binding site architecture is rigid and provides a stable scaffold for ligand binding. Conversely, several solvent-exposed loop regions displayed high flexibility, including those containing residues Ala247 and Val512 (highlighted by dashed lines). The pronounced flexibility of these loops, particularly those near the binding cavity entrance, may play a functional role in the initial recognition and accommodation of ligands.

To elucidate the molecular basis for their different binding dynamics, we analyzed the key interactions between each ligand and SGLT2 throughout the simulations (Figure 8D,E) using OpenMMDL (V1.1.1) with a contact threshold of 60% occupancy. The results showed that Isoonnin and Ononin bind to the protein through different combinations of interactions, including hydrophobic interactions, hydrogen bonds, and salt bridges. Isoononin establishes a stable binding pose anchored by a persistent network of interactions. Its isoflavone core is situated within a hydrophobic pocket formed by Lys154, Trp291, and Phe453 (Figure 8D). The stability of this hydrophobic engagement is reinforced by the formation of three persistent hydrogen bonds with Gln457, Thr87, and Asp454. The interaction timelines confirm that these contacts, particularly the hydrogen bonds, are maintained for a majority of the simulation (Figure 8F), providing a clear rationale for the low and stable RMSD observed for Isoononin. In contrast, Ononin demonstrates significant conformational adaptability. After reorienting to a new binding mode, it is stabilized by a different and more stable interaction network, despite undergoing a displacement of 4–5 Å between the initial docking pose (orange) and the equilibrated MD snapshot (cyan) (Figure 8E). While it forms hydrophobic contacts with residues like Val95, Phe98, Phe453 and Gln457, its stability in this new pose is secured by a diverse network of polar interactions. This network includes a salt bridge with the charged side chain of Lys154 and multiple hydrogen bonds with Lys154, Asn75, and Ser393. The timeline analysis shows that once this new interaction network is established, it remains consistent for the rest of the trajectory (Figure 8G).

**Figure 9:**
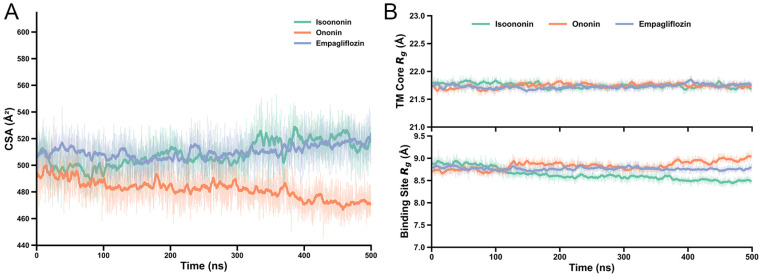
(**A**) Structural and interaction stability of SGLT2 complexes over 500 ns. Contact Surface Area (CSA) between inhibitors and protein. (**B**) Radius of Gyration (Rg) for the 14-TM helix bundle (top) and binding site residues within 3 Å (bottom). Solid lines represent moving averages; shaded regions denote raw trajectory data.

To quantify the stability of the inhibitor–protein interactions, we calculated the Contact Surface Area (CSA) and the Radius of Gyration (Rg) for all the systems. The CSA values for Isoononin and Empagliflozin remained relatively consistent, fluctuating between approximately 500 and 530 Å^2^, while Ononin exhibited a slightly lower but steady CSA of around 470–490 Å. Furthermore, the Rg analysis revealed negligible structural deviations throughout the 500 ns trajectories. Both 14-transmembrane helix bundle and the binding site residues (within 3 Å of the Empagliflozin) exhibited only minor fluctuations, indicates that the local architecture of the pocket remained stable upon inhibitor binding (Figure 9).

**Figure 10:**
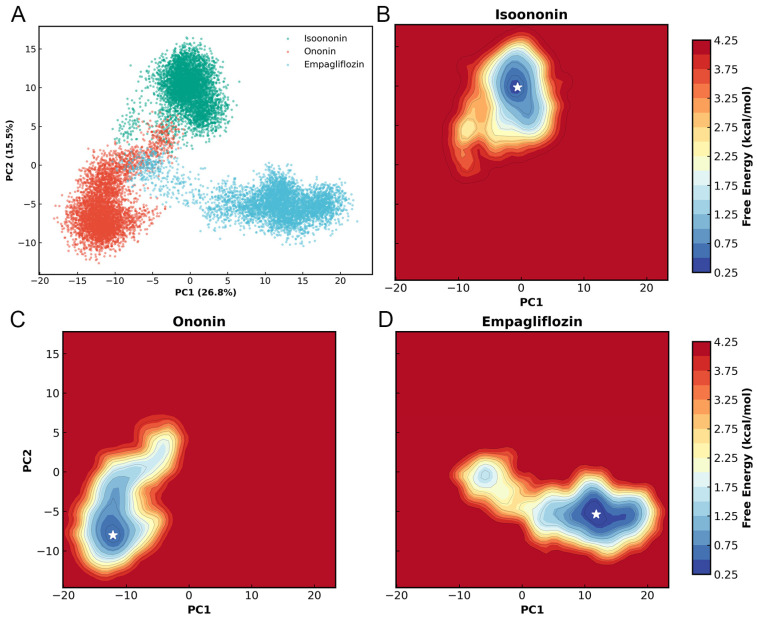
PCA and FEL of SGLT2 complexes. (**A**) 2D projection of concatenated trajectories onto PC1 and PC2 (variance in parentheses). (**B**–**D**) Individual free energy landscapes for the three complexes; white stars indicate global energy minima.

To characterize the essential dynamics of the SGLT2 complexes, Principal Component Analysis (PCA) was performed on the Cα atoms of the 14-helix bundle using a concatenated trajectory of all three systems. As shown in Figure 10, the first two principal components (PC1 and PC2) accounted for 26.8% and 15.5% of the total conformational variance, respectively, representing 42% of the total motions. The projection of the trajectories onto the PC1/PC2 subspace revealed three distinct clusters corresponding to the different ligand-bound states. While Isoononin, Ononin, and Empagliflozin occupy unique regions, it indicates inhibitor-specific influences on the protein’s dynamics.

Furthermore, Free Energy Landscapes (FEL) were constructed based on the PC1 and PC2 coordinates. Each landscape exhibits a well-defined and energy basin, with the global energy minimum for each complex marked by a white star. For the Isoononin and Ononin, the protein resides in a single energy well, indicating restricted conformational motion and good stability in protein. Meanwhile, the Empagliflozin complex explores a slightly broader energy basin, which might reflecting a degree of localized flexibility while remaining within a stable equilibrium state.

### 2.8. MMPBSA

To quantify and compare the binding affinities, we calculated the binding free energies (ΔGbind) for the Isoononin, Ononin, and FDA-approved drug Empagliflozin complexes using the MM-PBSA method extracted from the stable 500 ns MD trajectories. As summarized in Table 5, the ΔGbind values of Isoonnin and Ononin were −21.0 kcal/mol and −25.8 kcal/mol, respectively, indicating that Ononin has a slightly stronger binding affinity to SGLT2, albeit weaker than that of the clinical inhibitor Empagliflozin. The calculated absolute values −31.9 kcal/mol for Empagliflozin, are more negative than experimental values (−11.6 kcal/mol for Empagliflozin) because the configurational entropy change was not explicitly included, the relative ranking remains highly consistent with experimental potency [46,47]. Our result correlates with the more extensive network of polar interactions, including a salt bridge and hydrogen bonds, observed for Ononin in the structural analysis.

An analysis of the energy components revealed that van der Waals forces and nonpolar solvation energy are the primary energetic drivers for all three compounds, rather than polar contributions. This suggests that binding is thermodynamically dominated by good shape complementarity and a significant hydrophobic effect. Despite this, polar interactions may play an anchoring role and contribute to optimizing the favorable binding pose.

## 3. Discussion

SGLT2 inhibitors represent a novel class of medications for diabetes management. They function through a unique mechanism that does not depend on insulin for the regulation of blood glucose levels, rendering them compatible with existing hypoglycemic treatments, including insulin [48,49]. This characteristic positions SGLT2 inhibitors as an excellent option for the ongoing management of individuals with T2DM. Recent research suggests that these inhibitors also confer protective benefits for the heart and kidneys, significantly reducing the risk of cardiovascular events and slowing the progression of renal disease [19,50]. Overall, patients generally tolerate SGLT2 inhibitors well. However, some drawbacks are associated with their use, including the potential for urinary tract infections and ketoacidosis, and certain individuals may experience complications related to low blood pressure or decreased blood volume [51,52,53]. The investigation of new SGLT2 inhibitors is currently a significant area of scientific inquiry.

This study enhances the generalization performance of the model through optimized feature selection. In previous research, the random forest model demonstrated the best performance, achieving an accuracy of 0.74 and an AUC of 0.93 [34]. The model presented in this study significantly outperforms previously reported methods across all evaluation metrics (Figure 5 and Table 1), indicating that our model possesses higher predictive accuracy and reliability in the screening of SGLT2 inhibitors [34]. The integration of multi-source molecular fingerprint features with machine learning is crucial for the reliability of the predictive results. In the initial screening, most compounds were supported by only one to two fingerprint models. Therefore, this study increases the confidence of candidate compound predictions by integrating the predictive results from four different molecular fingerprint models. In the study, we employed models with slightly superior performance, namely LightGBM and RF, for the prediction of compound activity (Figure 5A,B), which led to the identification of 44 potential inhibitors. Among the eight MFH herbs we examined, six were found to contain potential SGLT2 inhibitors (Figure 6). Notably, Isoononin, derived from Gancao, and Oninin, derived from Huangqi, Gegen and Gancao exhibit significant SGLT2 inhibition potential and favorable drug-like properties.

Molecular docking studies demonstrate that both Isoononin and Ononin can effectively bind to the active pocket of SGLT2. The hydroxyl group of Isoononin forms hydrogen bonds with Tyr526 and Thr87, while the hydroxyl group of Ononin interacts with Tyr290, His80, and Gln457 through hydrogen bonding (Figure 7B,C). Additionally, both compounds establish hydrophobic interactions with the amino acid residues Phe98 and Phe453 (Figure 7B,C). Notably, the hydrophobic interactions between Isoononin and Phe453, as well as between Ononin and both Phe98 and Phe453, are sustained throughout most of the MD simulations (Figure 8F,G). Previous research has highlighted the significance of Phe98 and Phe453 in recognizing substrates and the inhibitory action of SGLT2 [9]. Alterations in these residues can diminish the binding strength of SGLT2 inhibitors to the SGLT2 protein, thereby reducing their inhibitory efficacy [9]. These findings imply that Isoononin and Ononin exhibit comparable pharmacophore traits and mechanisms of action to known SGLT2 inhibitors, suggesting their potential as SGLT2 inhibitors. Given that Ononin maintains stable hydrophobic interactions with critical amino acids (Phe98, Phe453) and has a slightly lower ΔGbind than Isoononin, it is hypothesized that Ononin may exhibit a greater SGLT2 inhibitory capacity.

**Figure 11:**
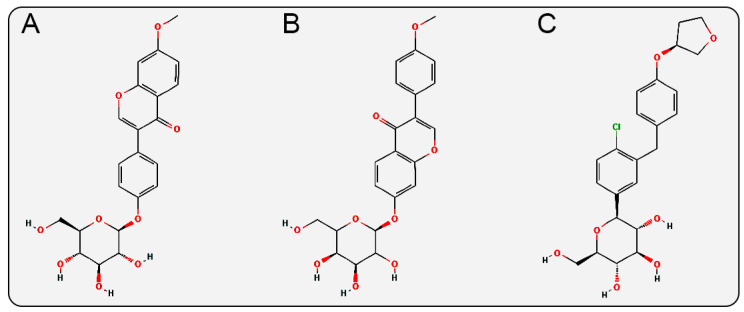
2D structures of (**A**) Isoononin, (**B**) Ononin, (**C**) Empagliflozin.

In the glycosidic ligand moiety of the inhibitors, Isoononin features a flavonoid structure containing a pyranone heterocycle, while Ononin has an isoflavone structure with a pyranone heterocycle. These two structures differ from the diphenylethylene structure of Empagliflozin (a biphenyl ring connected by a vinyl group, with a tetrahydrofuran heterocycle (Figure 11) and the phenylpropanoic acid structure of Phloridzin (a phenyl ring connected to a propanoic acid side chain, with multiple hydroxyl and keto carbonyl groups (Figure 1B)). The anti-inflammatory, antioxidant, and cardiovascular protective effects are also known pharmacological properties of flavonoid and isoflavonoid compounds [54,55,56,57], which may synergistically enhance their protective effects on cardiac and renal functions.

**Figure 12:**
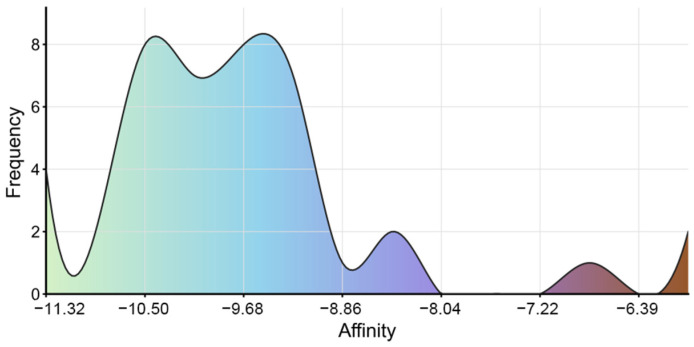
The binding energy distribution of 42 small molecule ligands with SGLT2.

Previous studies have identified Isoononin and Ononin as common compounds in traditional Chinese herbal medicine and leguminous plants, exhibiting various biological activities [58,59]. Ononin has shown significant regulatory effects on inflammation and various diseases, such as diabetic nephropathy, obesity, acute myocardial infarction, Alzheimer’s disease, cerebral ischemia–reperfusion, osteoarthritis, and tumors [58]. Research indicates that Ononin alleviates streptozotocin-induced diabetic nephropathy in rats by reducing oxidative stress and inflammatory markers [60]. On the other hand, Ononin exerts potential therapeutic effects in obesity and related metabolic diseases by inhibiting PPARγ-mediated anti-lipogenesis activity and reducing lipid accumulation in human adipocytes [61]. In terms of anti-tumor effects, Ononin inhibits the proliferation and migration of tumor cells and induces apoptosis by suppressing the aberrant activation of pathways such as EGFR-Erk1/2, PI3K/Akt/mTOR, MEK/ERK, and JNK/ERK/p38 [58,62]. Conversely, Isoononin, as an isomer of Ononin, has relatively limited research on its pharmacological activity. However, preliminary evidence suggests it possesses anti-inflammatory and anti-tumor potential [59,63]. These pharmacological characteristics expand the application prospects of Isoononin and Ononin in metabolic diseases. Additionally, we conducted molecular docking analysis on 42 compounds, excluding Isoononin and Ononin. The results indicated that all 42 compounds exhibited good binding affinity with SGLT2, with binding energies below −5.0 kcal/mol (Figure 12). The visualization results of the molecular docking are shown in Supplementary File S5. Although these compounds performed poorly in preliminary drug-likeness and ADME/T analyses, most of them had binding energies with SGLT2 ranging from −8.0 to −11.0 kcal/mol. This high-affinity binding suggests that they may still serve as potential lead compounds for structural optimization.

## 4. Materials and Methods

### 4.1. Data Collection and Preprocessing

Data on the biological activity and structural characteristics of SGLT2 inhibitors were sourced from the ChEMBL database (Target ID: CHEMBL3884) [64]. This collection features the quantitative active potency (IC_50_ values) for 2313 small molecules targeting SGLT2. The biological activity information was filtered to include only the standard activity type ‘IC_50_’, measured in nanomoles (nM). Any entries with missing data or duplicate compounds were removed, along with those that had invalid SMILES representations. Furthermore, 2000 decoy compounds were sourced from the DUD-E database and added to the inactive dataset. For these decoy compounds, IC_50_ values were randomly assigned to exceed 1000, ensuring they were distinctly categorized as inactive. The RDKit cheminformatics toolkit [65] was then employed to standardize the SMILES representations across the dataset. This standardization process involved preserving the largest organic fragment by molecular weight, eliminating salts and counterions, and normalizing protonation states, resulting in canonical SMILES that provide a unique and reproducible string representation for each chemical structure.

Following the previously outlined procedures, a collection of 1467 distinct SGLT2 inhibitors was assembled, each containing details on structure and bioactivity. To improve the numerical consistency of the ML models, the IC_50_ values underwent a negative logarithmic transformation, leading to the calculation of pIC_50_ = −log_10_(IC_50_[M]), with pIC_50_ values represented in moles. A classification threshold of 6.0 for pIC_50_ was established, which equates to an IC_50_ of 1000 nM. This benchmark is commonly accepted in pharmacological studies [66,67,68]. Compounds with pIC_50_ values of 6.0 or higher were classified as active (Class 1), while those with lower values were labeled as inactive (Class 0). Utilizing this criterion, 1317 compounds were identified as active, whereas 2150 were classified as inactive. The active and inactive compounds used in this study are stored in Supplementary File S1.

### 4.2. Molecular Fingerprint Generation

Molecular fingerprints provide a digital format for chemical data by breaking down the molecular architecture of compounds into distinct substructures or pathway patterns, which are subsequently converted into fixed-length binary strings [69]. These fingerprints are expressed as binary vectors, where the presence of certain features is indicated by a 1 and their absence by a 0 [69]. In this research, four commonly utilized types of molecular fingerprints are employed as input descriptors for the training and assessment of ML models: MACCS, RDKit, ECFP4, and TT [70,71,72]. All fingerprints are produced from canonical SMILES representations, utilizing the RDKit toolkit (V2020).

### 4.3. Compound Similarity Analysis

In order to assess the chemical variety within the selected dataset, we computed the Tanimoto similarity coefficient for pairs of compounds utilizing the 166-bit MACCS [73]. This coefficient is a widely recognized measure in cheminformatics for assessing molecular similarity, especially effective for evaluating binary fingerprints. The formula for calculating the Tanimoto coefficient is presented in Formula 1, where ‘a’ and ‘b’ indicate the total count of 1 s in the fingerprints of Compound A and Compound B, respectively, while ‘c’ signifies the number of shared features between the two compounds.

(1) $$\mathrm{Tanimoto}=\frac{c}{a+b\;-\;c}$$

### 4.4. Compound Feature Selection

To improve the model’s ability to generalize an minimize overfitting, we conducted feature selection on four fingerprint types by applying ‘low variance’ and ‘high correlation’ filters. Initially, we discarded descriptors with a variance below 0.01 to remove variables that offered little discrimination. Next, we computed the Pearson correlation coefficient to identify and eliminate features with high correlation (r > 0.85), addressing the problem of multicollinearity. Following this optimization process, the feature count for the MACCS decreased from 166 to 111, the RDKit from 1024 to 1018, the ECFP4 from 1024 to 813, and the TT from 1024 to 449. This refined feature set delivers a more efficient and information-rich input for ML models, enhancing both training efficiency and predictive accuracy.

### 4.5. Dataset Segmentation

To guarantee the robustness and generalizability of the model assessment, the dataset was randomly partitioned into training and testing subsets with a 70:30 distribution through a stratified sampling approach. The training subset is employed for model development and hyperparameter optimization, whereas the testing subset is used to evaluate the model’s performance on new, unseen data. This partitioning is conducted three times, resulting in three unique training/testing pairs (train_1_/test_1_, train_2_/test_2_, train_3_/test_3_). The 3 separate training-testing datasets can be consulted in Supplementary File S2. Each division preserves a uniform ratio of active to inactive compounds (Table 6). Molecular fingerprints were generated in four distinct formats for both the training and testing subsets, culminating in 12 separate training-testing datasets that facilitate the assessment of how various fingerprint characteristics and data divisions influence model efficacy.

### 4.6. Model Construction

The research utilizes several algorithms, including LightGBM, RF, XGBoost, DNN, Least Absolute Shrinkage and Selection Operator (LASSO) regression, and K-Nearest Neighbor (KNN). Various fingerprint descriptors serve as input features, while the output consists of binary classification labels for activities. Each model is trained with a consistent random seed (random_state = 42). To enhance reliability, hyperparameter tuning is conducted using a combination of grid search techniques and five-fold cross-validation, with performance verified on an independent validation set. The parameter configurations for the six ML algorithms utilized in this research are detailed as follows, while the remaining parameters are kept at their default settings.

#### 4.6.1. LightGBM Model

The LightGBM model relies on the lightgbm environment library (V4.4.0), with the parameter search space including the number of decision trees (100, 200), learning rate (0.01, 0.1), number of leaf nodes (31, 63), maximum decision tree depth (3, 5, −1), minimum number of samples (20, 50), sample sampling rate (0.8, 0.9, 1.0), and feature sampling rate (0.8, 0.9, 1.0).

#### 4.6.2. RF Model

The RF model is executed using the scikit-learn program (V1.6.1). The parameters for the RF model are set as follows the number of decision trees (10, 30, 50, 70, 100), maximum number of leaf nodes (20, 50, 100), and maximum number of features (1, 10, 20).

#### 4.6.3. XGBoost Model

The XGBoost model is implemented using the xgboost package (V2.1.4). The hyperparameter grid search encompasses the learning rate (0.1, 0.01), the number of weak classifiers (10, 50), the maximum tree depth (5, 10, 15), the sample sampling rate (0.5, 1.0), and the feature sampling rate (0.5, 1.0).

#### 4.6.4. DNN Model

The DNN model is developed and trained utilizing the Tensorflow package (V2.15). The DNN adopts a three-layer architecture consisting of ‘input-hidden-output’. The input layer receives normalized molecular fingerprint features. The hidden layer comprises two fully connected layers with 256 and 128 neurons, respectively, with a batch normalization layer and a dropout layer added after each fully connected layer. The output layer produces binary classification probabilities via a sigmoid function. The hyperparameter settings include: Dropout rates (0.2, 0.5), learning rates (0.0001, 0.001), batch sizes (32, 64), maximum training epochs (20), along with an early stopping strategy (patience = 5).

#### 4.6.5. KNN Model

The KNN model is implemented using the scikit-learn library (V1.6.1). Key parameters consist of the neighbor count the number of nearest neighbors (3, 5, 7, 9, 11), the method of weight calculation (uniform, distance), and the type of distance metric (Manhattan distance, Euclidean distance).

#### 4.6.6. LASSO Model

The LASSO model is implemented using the scikit-learn library (V1.6.1). The hyperparameter optimization focuses on the regularization strength C (0.001, 0.01, 0.1, 1, 10, 100) while also examining whether to fit the intercept term (including both True and False parameters).

### 4.7. Evaluation Metrics

Metrics used to assess the quality of a model encompass Precision, Sensitivity, Specificity, F1-score, Accuracy, Matthews Correlation Coefficient (MCC), and Youden’s Index [74]. Furthermore, the AUC serves as a key indicator of the model’s overall ability to distinguish between classes, making it an essential factor in quality evaluation [75]. The calculations for these metrics are detailed below (Table 7).

### 4.8. TCM Compound Collection and SGLT2 Inhibition Activity

We selected several MFH herbs for the treatment of diabetic heart disease (DHD) as sources of compounds, including Huangqi (Astragali Radix), Maidong (Ophiopogonis Radix), Danggui (Angelicae Sinensis Radix), Fuling (Poria Cocos), Gegen (Puerariae Lobatae Radix), Dihuang (Rehmanniae Radix), Honghua (Carthami Flos), and Gancao (Glycyrrhizae Radix). These herbs were identified through our preliminary analysis of the literature related to DHD and are commonly used due to their well-defined pharmacological effects and application bases. We compiled the compounds from these herbs using the TCMbank database (https://tcmbank.cn/ [76]), creating a repository of active compounds from TCM. By applying the same descriptors and preprocessing methods utilized for the training data, we derived molecular fingerprints for all compounds to ensure consistency within the feature space.

In our pursuit of novel SGLT2 inhibitors, we conducted activity assessments on the compounds sourced from a curated collection of components derived from MFH herbs. Each compound was assigned an individual score based on models developed from four distinct fingerprint types. Those identified as ‘highly active’ by all four models were selected for further investigation.

### 4.9. Drug-like Analysis and ADME/T Prediction

Utilizing the principles of “Lipinski” and “Verber”, the SwissADME platform (https://www.swissadme.ch/ [77]) (accessed on 30 October 2025) was employed to assess the drug-like characteristics of the chosen candidate compounds. This assessment included criteria such as molecular weight (MW ≤ 500 Da), hydrogen bond donors (HBD ≤ 5), hydrogen bond acceptors (HBA ≤ 10), lipophilicity (LOGP ≤ 5), rotatable bonds (RB ≤ 10), and polar surface area (TPSA ≤ 140). Additionally, the ADME/T (Absorption, Distribution, Metabolism, Excretion, and Toxicity) properties were predicted using three webservers: pkCSM (https://biosig.lab.uq.edu.au/pkcsm/ [78]) (accessed on 30 October 2025), AdmetSAR 2.0 (http://lmmd.ecust.edu.cn/admetsar2 [79]) (accessed on 30 October 2025) and ADMETlab 2.0 platform (https://admetmesh.scbdd.com/ [72]). Critical factors such as intestinal absorption rates, potential for CYP450 enzyme inhibition, hepatotoxicity, carcinogenicity, and AMES toxicity were thoroughly analyzed.

### 4.10. Y-Randomization

Y-randomization validation is a widely utilized technique for assessing the reliability of models, focusing on determining if there is a random association between the dependent and independent variables. During this validation, the input features (X) remain constant while the training set labels (Y) are shuffled randomly. The model is then retrained on this modified dataset, maintaining the same hyperparameters as the initial model, and the AUC and accuracy of the randomized model are evaluated against the original test set. To evaluate the model’s performance, we resampled 75% of the compounds in the training set and conducted 500 randomization tests, thereby creating a distribution of performance metrics for the random model.

### 4.11. Molecular Docking

A flexible docking method was utilized for molecular docking. The three-dimensional configurations of the compounds Isoononin (CID: 5318619) and Ononin (CID: 44257215) were retrieved from the PubChem database (https://pubchem.ncbi.nlm.nih.gov), while the crystal structure of the SGLT2 protein (hSGLT2, PDB ID: 7VSI) was obtained from the RCSB Protein Data Bank (https://www.rcsb.org). The active site was designated as a region extending 20.0 Å from the coordinates of the endogenous ligand center (x = 38.292, y = 50.143, z = 46.481). The docking of the single-chain receptor protein with the active ligand components was carried out using AutoDock Vina (V1.1.2). The docking calculations were performed with a genetic algorithm, employing default settings for all run options. After the docking process, the stabilized complexes were visualized with PyMOL (V2.3) to analyze the interactions between the compounds and the protein.

### 4.12. Molecular Dynamics Simulations

All-atom MD simulations were performed to elucidate the dynamic binding behavior and interaction mechanisms of Isoononin and Ononin with the human sodium-glucose cotransporter 2 (hSGLT2, PDB ID: 7VSI), using the highest-scoring docking poses as the starting configurations. The simulation systems were constructed using the CHARMM-GUI web server [80], where each protein-ligand complex was embedded in a 1-palmitoyl-2-oleoyl-sn-glycero-3-phosphocholine (POPC) lipid bilayer. The Amber ff19SB force field [81] was employed for the protein, and Lipid21 [82] for the lipids, while the small molecules (Isoononin, Ononin and Empagliflozin) were parameterized using the General Amber Force Field (GAFF2) with partial charges assigned by the Antechamber module. We appiled OpenMM (V8.0) [83] for all the molecular dynamics simulations, each system was first minimized for 5000 steps, followed by a six-stage equilibration where constraints on the lipids and protein were gradually removed. A 150 ns production MD simulation was then performed in the NPT ensemble with periodic boundary conditions. The simulation was maintained at 310 K (Langevin thermostat) and 1 atm (Monte Carlo anisotropic barostat). The Particle Mesh Ewald (PME) method was utilized for long-range electrostatic calculations. A 12 Å cutoff was applied for non-bonded interactions, including van der Waals forces. The integration time step is 2 fs, with bonds to hydrogen constrained by the SHAKE algorithm. The resulting MD trajectories were analyzed using VMD (V1.93), MDAnalysis (V2.10), and OpenMMDL (V1.1.1) [84]. Specifically, OpenMMDL was employed to investigate binding modes and track the time series of key interactions throughout the simulation. For binding mode identification, a threshold of 60% was selected to prioritize the most persistent and stable interactions. The global compactness of the protein throughout the simulation was monitored via the Radius of Gyration (Rg) and the Contact Surface Area (CSA) of the inhibitor within the binding pocket were investigated by calculating the Solvent Accessible Surface Area (SASA) using the Shrake-Rupley algorithm as implemented in MDTraj with following equation.

(9) $$CSA=\frac{1}{2}(SASA_{protein}+SASA_{inhibitor}-SASA_{complex})$$

### 4.13. Principal Component Analysis (PCA) Free Energy Landscape (FEL)

Principal Component Analysis (PCA) was performed to characterize the essential dynamics of the three inhibitor-bound systems. To establish a unified coordinate system for direct comparison, all three trajectories were concatenated to define a shared essential subspace. In this analysis, MDAnalysis was employed to align the Cα atoms of the core 14-helix bundle, filtering out global motions and suppressing the noise from non-essential fluctuations. Following covariance matrix construction and diagonalization via Scikit-learn, the trajectories were projected onto the first two principal components (PC1 and PC2) to visualize the essential motions of the protein-inhibitor complexes.

To further investigate the thermodynamic stability and conformational transitions of SGLT2 induced by different ligands, free energy landscapes (FEL) were constructed. The FEL was projected onto the first two principal components (PC1 and PC2) derived from the shared PCA calculations. The Gibbs free energy was calculated using the following equation:

(10) $$G(PC_{1},PC_{2})=-k_{B}T\ln P(PC_{1},PC_{2})$$

where is the Boltzmann constant, T is the simulation temperature (310 K), and P (PC_1_,PC_2_) represents the joint probability distribution of the protein system along the two principal components. The probability density was estimated using a binning grid. The resulting energy values were normalized such that the global minimum of each system was set to 0 kcal/mol.

### 4.14. MMPBSA

The binding free energy of each protein-ligand complex was calculated from MD simulation trajectories using the molecular mechanics Poisson-Boltzmann surface area (MM/PBSA) method. For these calculations, snapshots were extracted from the final 110 ns of the trajectories (40 to 150 ns) at 2 ns intervals. A heterogeneous dielectric implicit membrane model was employed to accurately recapitulate the membrane environment in the free energy calculations. The MMPBSA.py module within the Amber software suite (V2025) was used for this analysis [85,86].

## 5. Conclusions

This study developed an integrated virtual screening pipeline for SGLT2 inhibitors and identified 44 potential SGLT2 inhibitors from a compound library derived from eight selected MFH herbs. Among these, Isoononin (from Gancao) and Oninin (from Huangqi, Gegen and Gancao) exhibit strong SGLT2 inhibitory potential and favorable drug-like properties. Our study provides candidate molecules for the development of novel SGLT2 inhibitors and offers new scientific support for the application of MFH herbs in the prevention and treatment of chronic metabolic diseases. Subsequent in vitro and in vivo pharmacodynamic experiments are necessary to verify the inhibitory activity of Oninin and Isoononin on the SGLT2 protein and their hypoglycemic effects, as well as to further explore their metabolic stability, toxicological properties, and mechanisms of action.

## Figures and Tables

**Table 1:** Performance evaluation of the best models of LightGBM and RF on the training set and testing set.

Model	Training Set					Testing Set				
	Accuracy	Precision	Sensitivity	Specificity	Youden	Accuracy	Precision	Sensitivity	Specificity	Youden
RF										
MACCS	0.97197	0.93967	0.99025	0.96077	0.95102	0.96926	0.93112	0.99241	0.95511	0.94751
RDKit	0.97197	0.95241	0.97505	0.97009	0.94513	0.97406	0.95545	0.97722	0.97214	0.94935
ECFP4	0.97568	0.94563	0.99350	0.96476	0.95826	0.98079	0.95844	0.99241	0.97368	0.96609
TT	0.97444	0.95093	0.98372	0.96876	0.95248	0.97983	0.96985	0.97722	0.98142	0.95864
LightGBM										
MACCS	0.97032	0.93036	0.99676	0.95412	0.95088	0.96830	0.92689	0.99494	0.95201	0.94695
RDKit	0.97238	0.94712	0.98265	0.96610	0.94875	0.97502	0.96010	0.97468	0.97523	0.94992
ECFP4	0.97651	0.95117	0.98917	0.96876	0.95793	0.97502	0.94891	0.98734	0.96749	0.95483
TT	0.97815	0.95635	0.98807	0.97207	0.96014	0.97214	0.95980	0.96709	0.97523	0.94232

Training/Testing set: Evaluation of model performance in the Training/Testing. Accuracy: Proportion of correctly predicted compounds. Precision: Proportion of true active compounds among predicted actives. Sensitivity: Proportion of true actives correctly identified. Specificity: Proportion of true inactives correctly identified. Youden Index: Sensitivity + Specificity − 1 (higher values indicate better classification performance).

**Table 2:** Drug-like analysis based on the “Lipinski” and “Verber” rules.

Name	MW (g/mol)	HBD	HBA	LogP	Num.Rotatable Bonds	TPSA (Å2)	Source
Ononin	430.41	4	9	0.97	5	138.82	Gancao
8-methoxy-5-o- 9-glucoside flavone	430.41	4	9	0.83	5	138.82	Gancao, Gegen
Isoononin	430.41	4	9	0.84	5	138.82	Gancao
Formononetin-7- glucoside	430.41	4	9	0.96	5	138.82	Huangqi, Gancao, Gegen

**Table 3:** Predicted ADME/T (absorption, distribution, metabolism, and excretion) properties of four high potential compounds.

TERM	Compounds			
	Ononin	8-methoxy-5-o- 9-glucoside flavone	Isoononin	Formononetin-7- glucoside
GI absorption	High	High	High	High
Pgp substrate	Yes	No	No	Yes
BBB permeant	No	No	No	No
CYP1A2 inhibitor	No	No	No	No
CYP2C19 inhibitor	No	No	No	No
CYP2C9 inhibitor	No	No	No	No
CYP2D6 inhibitor	No	No	No	No
CYP3A4 inhibitor	Yes	Yes	Yes	Yes

**Table 4:** Toxicity prediction of four high-potential compounds.

TERM	Compounds			
	Ononin	8-methoxy-5-o- 9-glucoside flavone	Isoononin	Formononetin-7- glucoside
hERG inhibition	Low	Low	Low	Low
Human hepatotoxicity	Low	Low	Low	Low
Drug induced liver injury	Medium	High	Medium	High
AMES toxicity	Low	Medium	Low	Medium
Carcinogenicity	Medium	Medium	Medium	High

**Table 5:** Summary of MM-PBSA calculations for estimating the binding free energy of Isoonnin, Ononin, and Empagliflozin to SGLT2. The energetic contributions include van der Waals (ΔE_vdw_), electrostatics (ΔG_ele,total_, including both gas-phase and polar solvation contributions), and nonpolar solvation (ΔG_cavity_, ΔG_disp_).

Compound	ΔG_bind_	ΔE_vdw_	ΔG_ele,total_	ΔG_cavity_	ΔG_disp_	ΔG_polar_	ΔG_nonpolar_
Isoononin	−21.0	−51.6	0.8	−37.8	67.7	0.8	−21.8
Ononin	−25.8	−54.1	−0.3	−41.3	69.9	−0.3	−25.5
Empagliflozin	−31.9	−60.5	−0.03	−43.7	72.3	−0.03	−31.87

**Table 6:** Number of compounds in datasets for ML models.

	Active	Inactive	Total
Train_1/2/3_	922	1504	2426
Test_1/2/3_	395	646	1041

**Table 7:** Model performance evaluation metrics.

Formula	Number
$\mathrm{Precision}=\frac{\mathrm{TP}}{\mathrm{TP}+\mathrm{FP}}$	(2)
$\mathrm{Sensitivity}=\frac{\mathrm{TP}}{\mathrm{TP}+\mathrm{FN}}$	(3)
$\mathrm{Specificity}=\frac{\mathrm{TN}}{\mathrm{TN}+\mathrm{FP}}$	(4)
$\mathrm{Accuracy}=\frac{\mathrm{TP}+\mathrm{TN}}{\mathrm{TP}+\mathrm{TN}+\mathrm{FP}+\mathrm{FN}}$	(5)
$F1\text{-}\mathrm{score}=\frac{2\mathrm{TP}\;}{2\mathrm{TP}+\mathrm{FP}+\mathrm{FN}}$	(6)
$\mathrm{MCC}=\frac{\mathrm{TP}*\mathrm{TN}\;-\;\mathrm{FP}*\mathrm{FN}\;}{\sqrt{\left(\mathrm{TP}+\mathrm{FP})(\mathrm{TP}+\mathrm{FN})(\mathrm{TN}+\mathrm{FP})(\mathrm{TN}+\mathrm{FN}\right)}}$	(7)
$Y\mathrm{ouden}=\frac{\mathrm{TP}}{\mathrm{TP}+\mathrm{FN}}+\frac{\mathrm{TN}}{\mathrm{TN}+\mathrm{FP}}-1$	(8)

True positives (TP) refer to the number of compounds correctly predicted as active, while true negatives (TN) indicate the number of compounds correctly predicted as inactive. False positives (FP) are the number of compounds incorrectly predicted as active, and false negatives (FN) are the number of compounds incorrectly predicted as inactive.

## Data Availability

The original contributions presented in this study are included in the article. Further inquiries can be directed to the corresponding authors.

## Supplementary Materials

The following supporting information can be downloaded at: https://www.mdpi.com/article/10.3390/ph19020246/s1, Supplementary File S1: The active and inactive compounds used in this study. Supplementary File S2: Three separate training-testing datasets used in this study. Supplementary File S3: The prediction results of two models for the compounds of MFH herbs. Supplementary File S4: Forty-four compounds with high SGLT2 inhibition potential were identified by LightGBM model and RF model. Supplementary File S5: Molecular docking visualization diagrams of forty-two compounds with SGLT2.

## Abbreviations

The following abbreviations are used in this manuscript:

SGLT2	Sodium-glucose co-transporter 2
MFH	Medicine food homology
ML	Machine learning
MD	Molecular dynamics
T2DM	Type 2 diabetes mellitus
TCM	Traditional Chinese medicine
LightGBM	Light Gradient Boosting Machine
RF	Random Forest
XGBoost	Extreme Gradient Boosting
DNN	Deep Neural Network
LASSO	Least Absolute Shrinkage and Selection Operator
KNN	K-Nearest Neighbor
ECFP4	Extended connectivity fingerprint radius 2
TT	Topological torsion fingerprints
MCC	Matthew’s correlation coefficient
AUC	Area under the ROC curve
