# Supplementary material for: Computational Discovery of Novel SGLT2 Inhibitors from Eight Selected Medicine Food Homology Herbs Using a Multi-Stage Virtual Screening Pipeline
Source: Pharmaceuticals (Basel). 2026 Jan 31;19(2):246. doi: 10.3390/ph19020246 (PMC12943391; doi:10.3390/ph19020246)

TCMBANKIN039049<br>Affinity = -5.777 kcal/mol	TCMBANKIN040896<br>Affinity = -5.816 kcal/mol
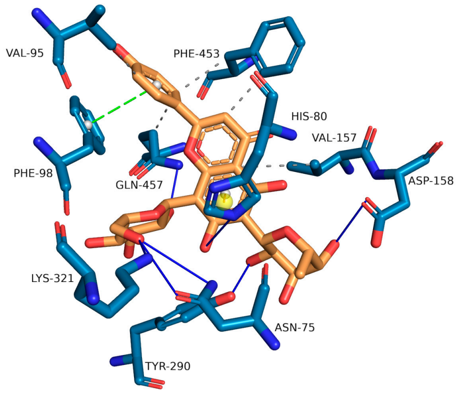	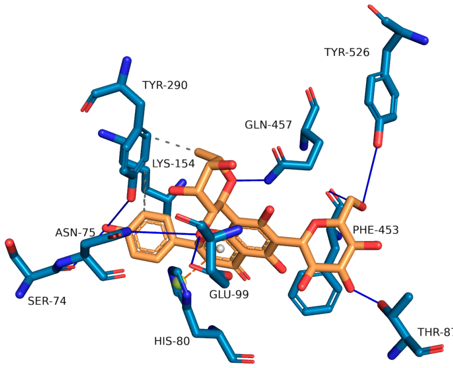
TCMBANKIN037352<br>Affinity = -6.879 kcal/mol	TCMBANKIN061276<br>Affinity = -8.63 kcal/mol
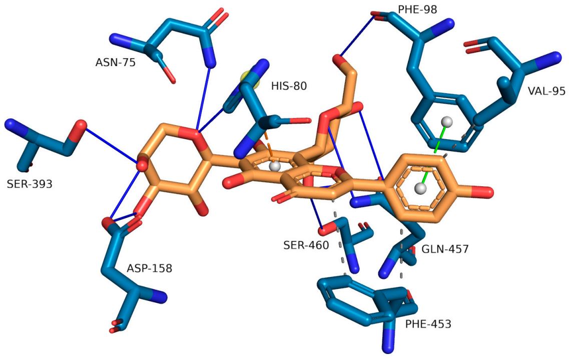	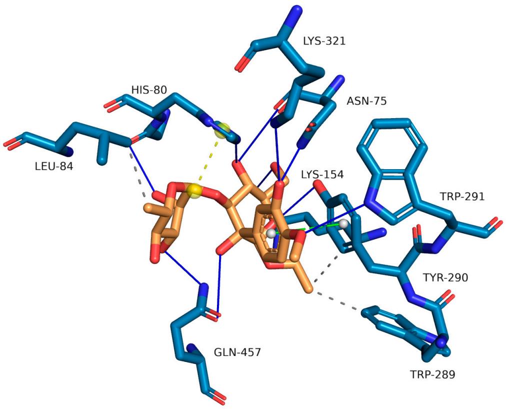
TCMBANKIN016632<br>Affinity = -8.645 kcal/mol	TCMBANKIN036837<br>Affinity = -8.726 kcal/mol
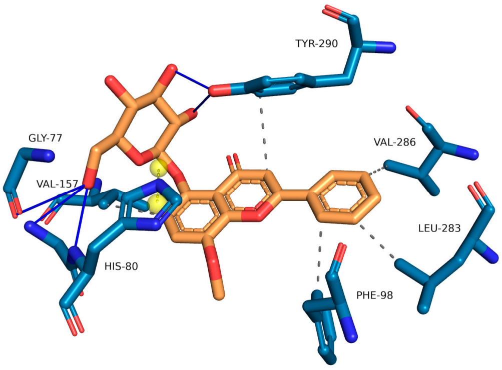	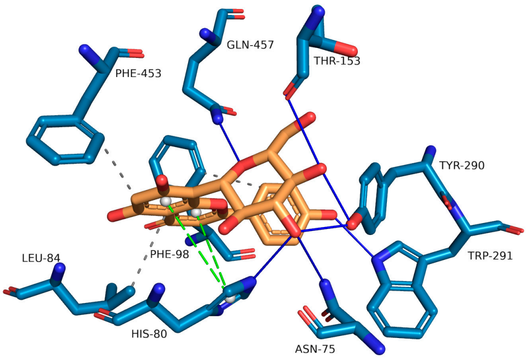
TCMBANKIN048960<br>Affinity = -9.07 kcal/mol	TCMBANKIN058478<br>Affinity = -9.146 kcal/mol
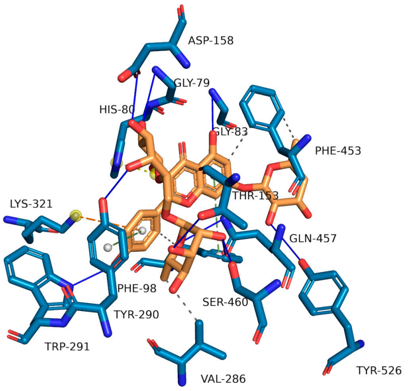	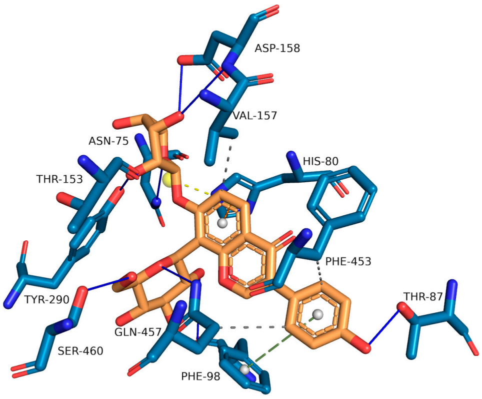

TCMBANKIN020726
Affinity = -9.169 kcal/mol

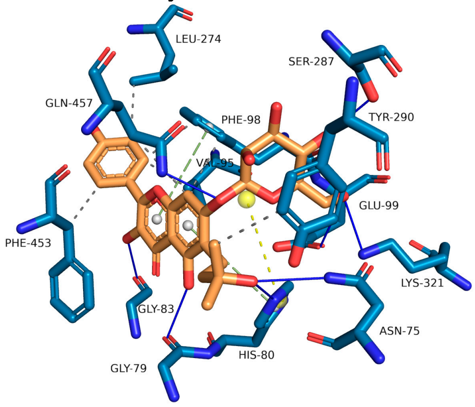

TCMBANKIN046408
Affinity = -9.215 kcal/mol

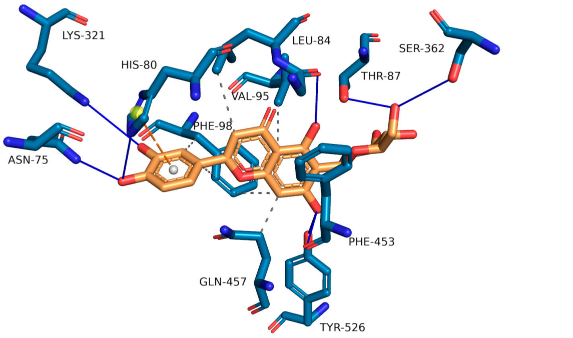

TCMBANKIN001649
Affinity = -9.257 kcal/mol

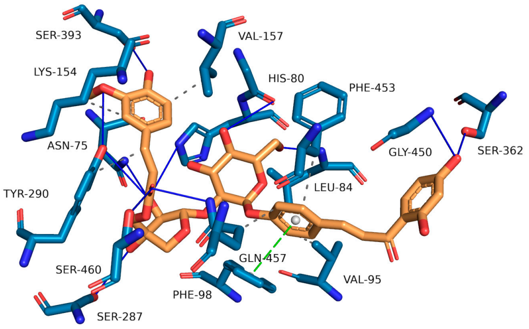

TCMBANKIN037869
Affinity = -9.276 kcal/mol

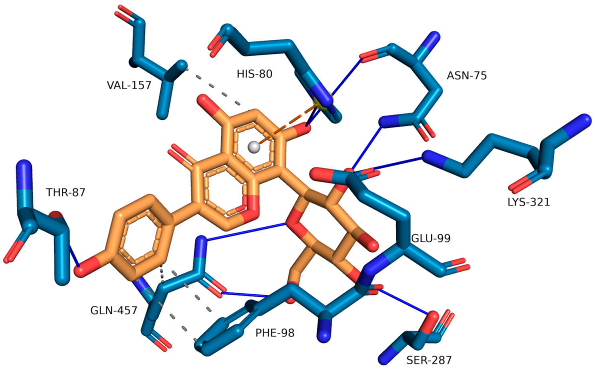

TCMBANKIN039940
Affinity = -9.412 kcal/mol

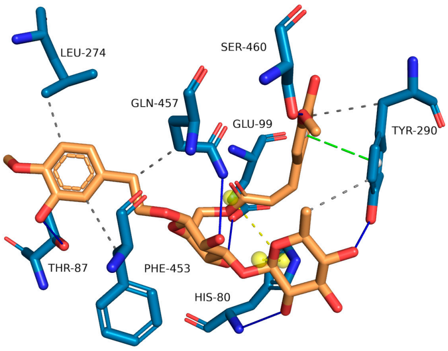

TCMBANKIN033269
Affinity = -9.498 kcal/mol

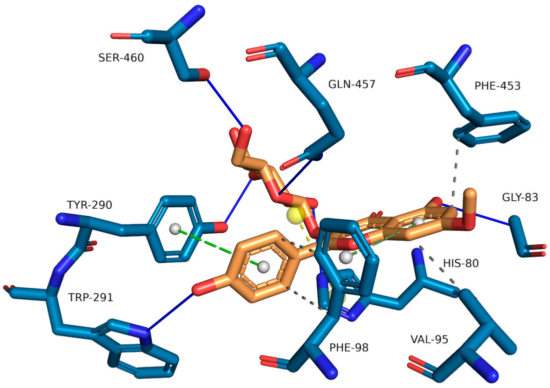

TCMBANKIN061438
Affinity = -9.591 kcal/mol

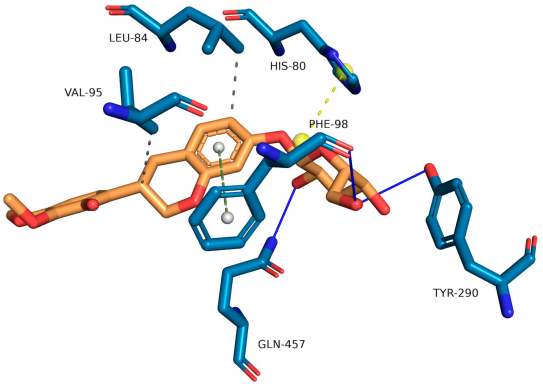

TCMBANKIN044055
Affinity = -9.623 kcal/mol

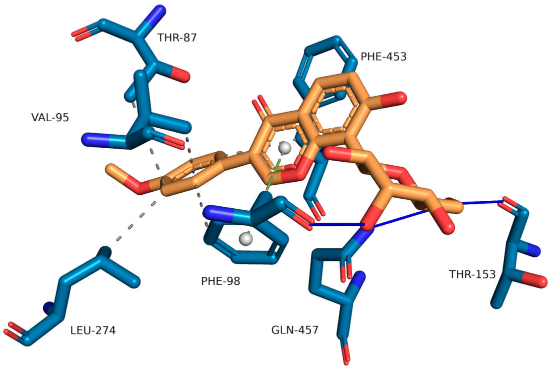

TCMBANKIN058215
Affinity = -9.664 kcal/mol

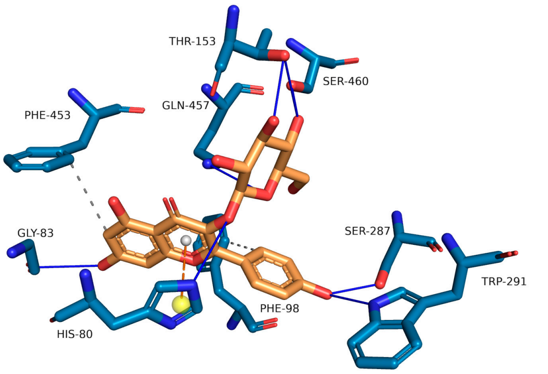

TCMBANKIN061911
Affinity = -9.667 kcal/mol

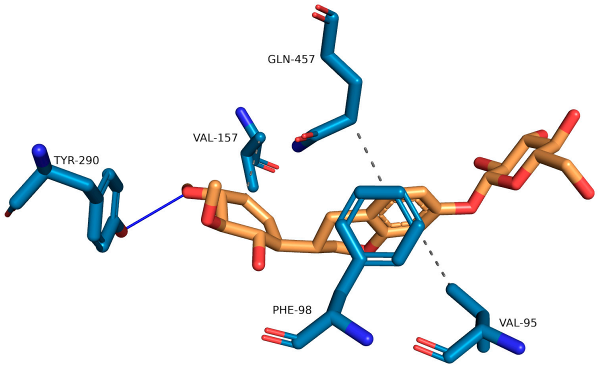

TCMBANKIN002008
Affinity = -9.827 kcal/mol

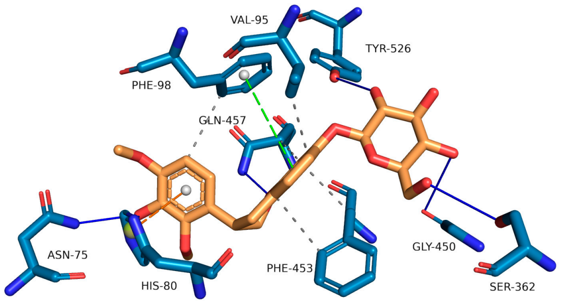

TCMBANKIN058230
Affinity = -9.859 kcal/mol

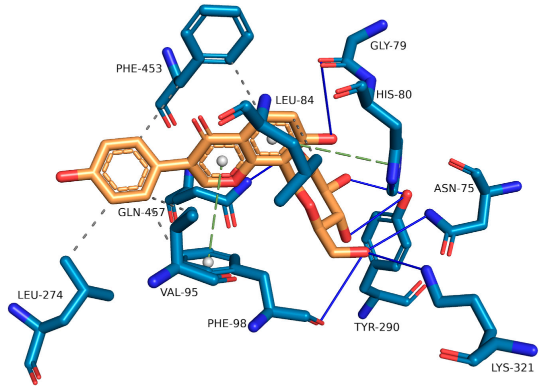

TCMBANKIN058216
Affinity = -9.882 kcal/mol

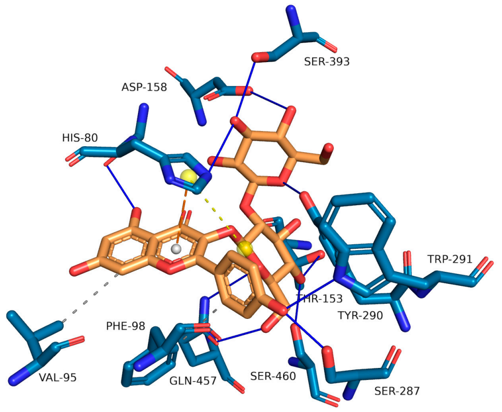

TCMBANKIN039736
Affinity = -9.903 kcal/mol

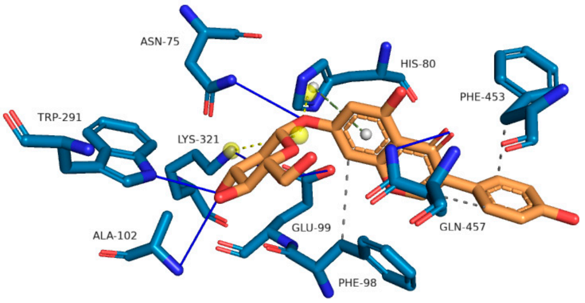

TCMBANKIN043473
Affinity = -10.09 kcal/mol

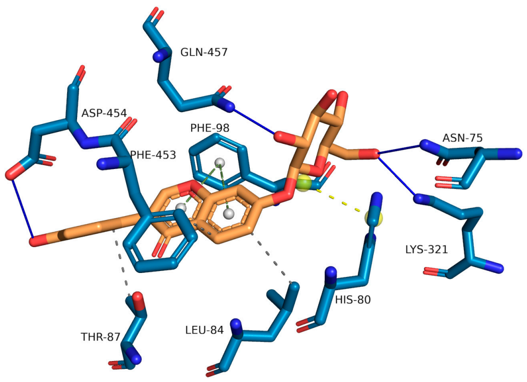

TCMBANKIN038308
Affinity = -10.1 kcal/mol

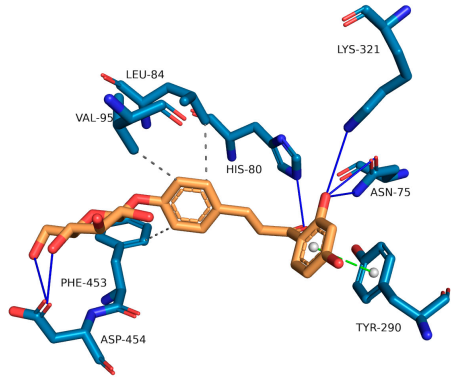

TCMBANKIN061600
Affinity = -10.11 kcal/mol

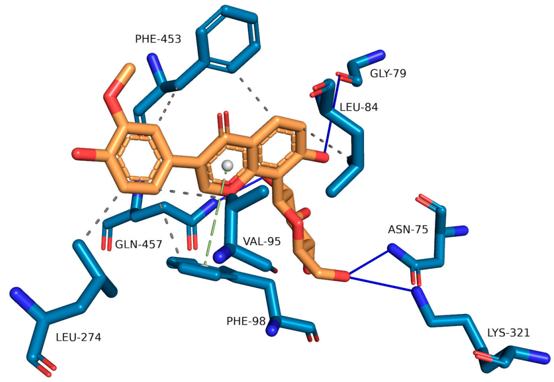

TCMBANKIN058193
Affinity = -10.16 kcal/mol

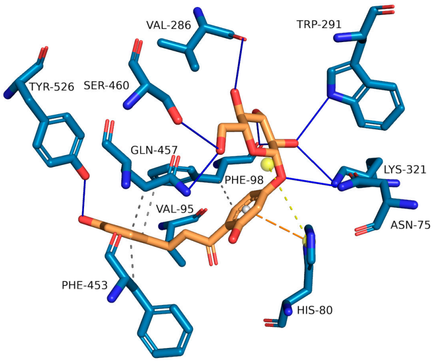

TCMBANKIN060176
Affinity = -10.17 kcal/mol

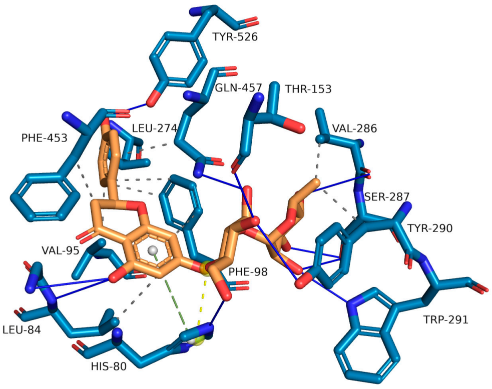

TCMBANKIN035749
Affinity = -10.19 kcal/mol

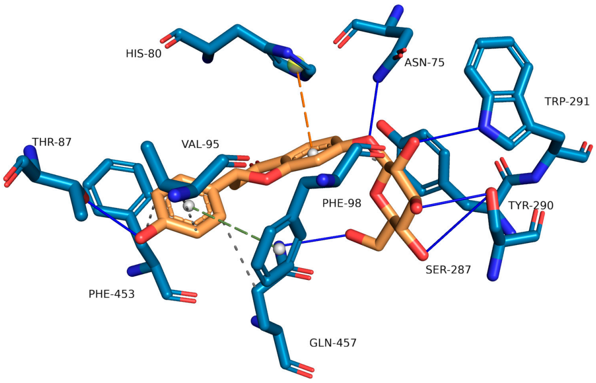

TCMBANKIN034279
Affinity = -10.33 kcal/mol

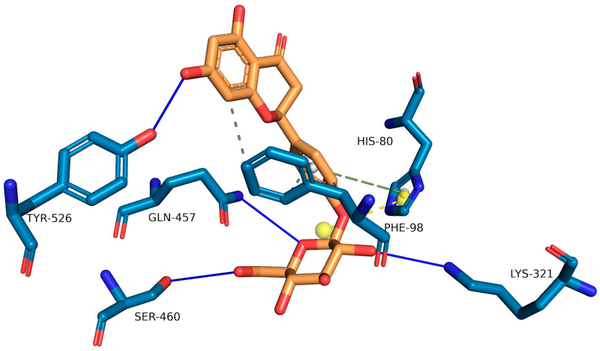

TCMBANKIN019108
Affinity = -10.34 kcal/mol

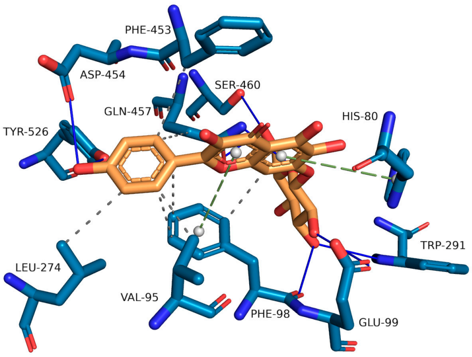

TCMBANKIN016653
Affinity = -10.36 kcal/mol

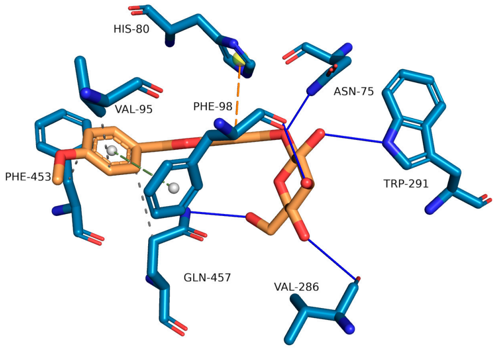

TCMBANKIN046782
Affinity = -10.42 kcal/mol

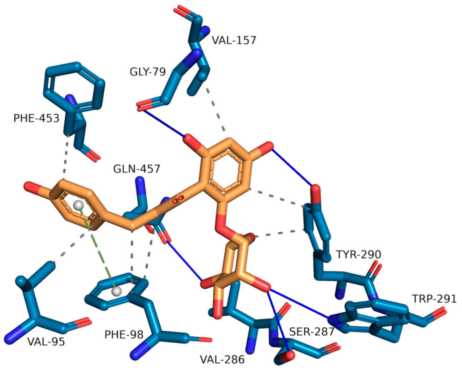

TCMBANKIN033202
Affinity = -10.51 kcal/mol

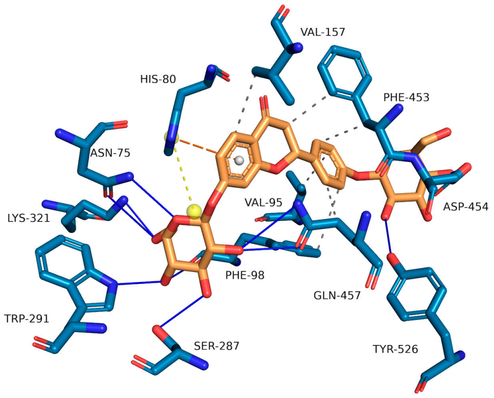

TCMBANKIN040788
Affinity = -10.53 kcal/mol

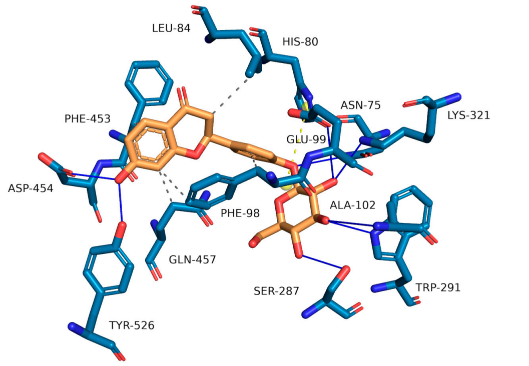

TCMBANKIN036796
Affinity = -10.55 kcal/mol

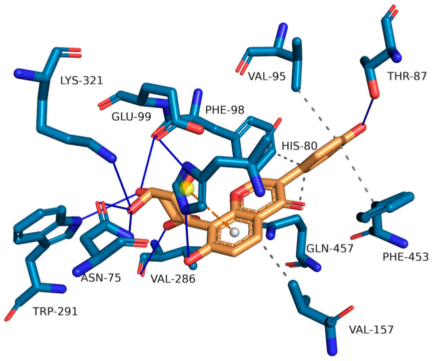

TCMBANKIN060262
Affinity = -10.56 kcal/mol

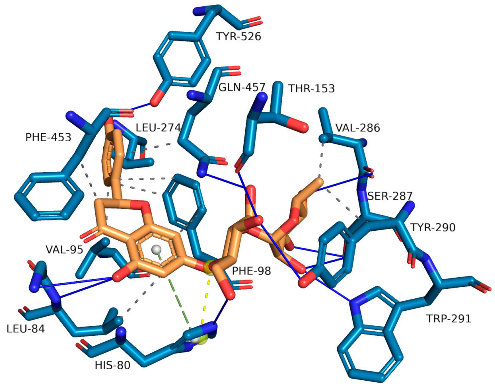

TCMBANKIN030208
Affinity = -10.77 kcal/mol

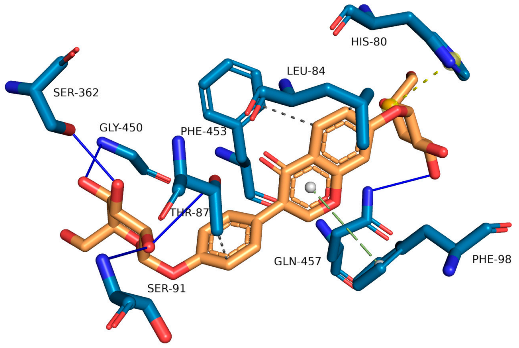

TCMBANKIN048910
Affinity = -10.79 kcal/mol

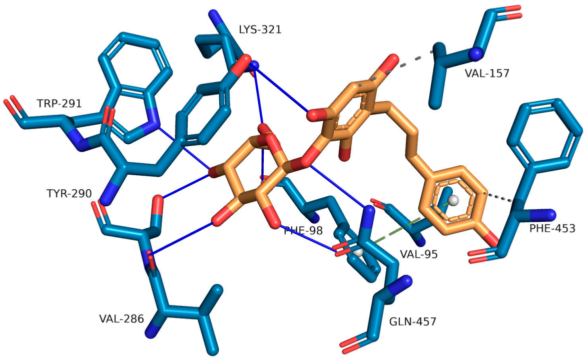

TCMBANKIN015934
Affinity = -11.17 kcal/mol

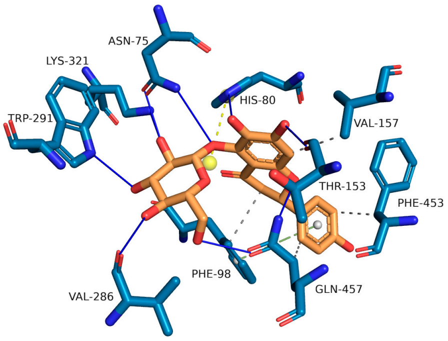

TCMBANKIN046374
Affinity = -11.2 kcal/mol

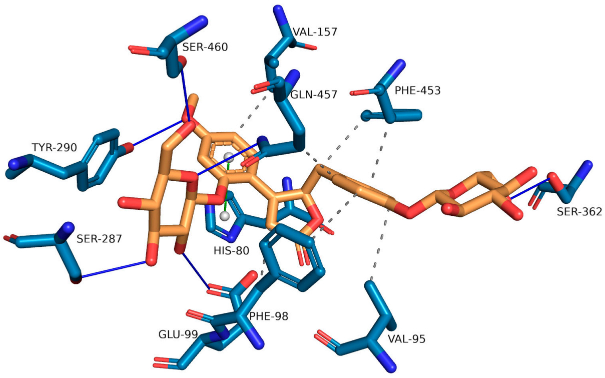

TCMBANKIN058474

Affinity = -11.2 kcal/mol

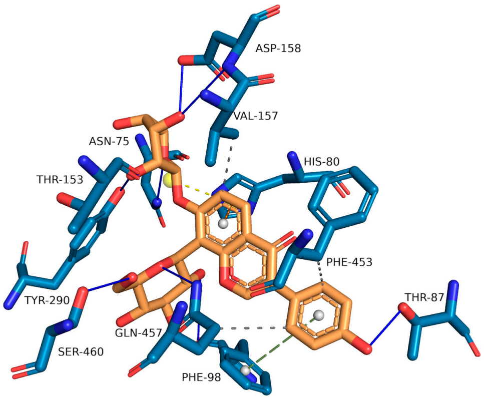

TCMBANKIN043983

Affinity = -11.53 kcal/mol

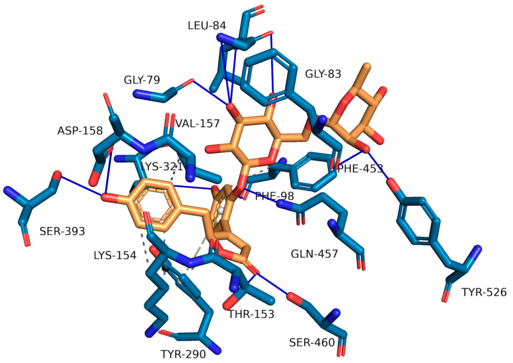

Supplement: Supplementary file 1 [file pharmaceuticals-19-00246-s001.zip › Supplementary file S5_Molecular docking visualization diagrams of forty-two compounds with SGLT2.pdf]
